# The Economic Impact of Managing Late Presentation of Developmental Dysplasia of Hip (DDH)

**DOI:** 10.5704/MOJ.1511.006

**Published:** 2015-11

**Authors:** RIM Anuar, HP Mohd-Hisyamudin, MH Ahmad, O Zulkiflee

**Affiliations:** Department of Orthopaedics, Penang Hospital, Georgetown, Malaysia

**Keywords:** Economic impact, late presentation, DDH

## Abstract

Delayed presentation of Developmental Dysplasia of Hip (DDH) comes with challenges in treatment as well as high surgical cost. Therefore the objective of this study is to quantify the economic impact of management of late presentation of DDH during a last 3-year period. We conducted a retrospective study with analysis of DDH cases managed between years 2012 to 2014. Early and late presentations of DDH were identified and cost management for both was estimated. Out of twenty-four DDH cases, thirteen cases fulfilled the inclusion criteria. All were female with majority of them presenting with unilateral DDH predominantly of the left hip. Most patients presented after age of six months and the principal complaint was abnormal or limping gait. The grand total cost for managing DDH during the three years period was USD 12,385.51, with 86% of the amount having been used to manage late presentation of DDH that was mostly contributed by the cost of surgery. We concluded that delayed presentation of DDH contributes heavily to high national expenditure. Early detection of DDH cases with systematic neonatal screening may help to minimize the incidence of the late presenting DDH and subsequently reduce the economic burden to the government.

## Introduction

The incidence of Developmental Dysplasia of Hip (DDH) -varies among countries. In Malaysia, it was reported as 0.7 to 12.2 per 1,000 live births^1-2^. The documented incidence of late presentation of DDH is alarming since almost half of DDH cases in India presented after one year of age and about 80% of cases in University of Malaya Medical Centre (UMMC) came only after age of four months^3-4^. In general, the management of the DDH - largely depends on the age of the children’s - first presentation to the orthopaedic surgeon. The early detection of DDH has better outcome and prognosis and the potential of being treated non-operatively, whereas in delayed presentation of DDH the majority of them are managed by surgical intervention.

The surgical options in managing the late presentation group depends mainly on age of the patients as well as the severity of the dysplastic hip at the time of presentation. The older child with severe hip problem may require more complex surgery involving bony procedures. These surgeries will come with a significant cost compared to non-operative treatment in early detection of DDH cases. A previous study did evaluate the economic benefit of neonatal screening in DDH^5^. As there is no recent data on cost estimation in managing late presentation of DDH, we conducted this study to quantify financial impact of management of patients with delayed presentation of DDH in our centre.

## Materials and Methods

We retrospectively analyzed patients with hip problems managed by the Paediatric Orthopaedic team from 2012 to 2014. The inclusion criterion was idiopathic DDH which presented early or delayed to our clinic with complete records and documentation in our data system. All nonidiopathic hip problems for instance syndromic child, spina bifida and sacral agenesis were excluded.

Cost was estimated for management of both non-operative and operative patients. For the non-operative treatment of early detection of DDH, the cost included ultrasound screening, Pavlik Harness and abduction splint^6^ (Table I).

**Table I:** Cost of Early Detection of DDH

**Item**	**Cost (USD)**
Ultrasound Screening	26.74
Pavlik Harness	53.48
Abduction Splint	13.37

For the operative group, we quantified the surgical cost depending on the type of surgery. The surgical costs included operation theatre cost, surgeon’s and anaesthetist’s fees, as well as admission costs. Operation theatre cost was based on the materials used in the DDH operation^7^ (Table II). Surgeon and anaesthetist’s fees were estimated based on the Full Paying Patient (FPP) Guideline 2007 Modified 2015 Ministry of Health^8^ (Table III). The operation theatre cost was estimated at rate of USD53.48 per hour.

**Table II:** Operation Theatre Fee (materials)

**Item**	**Cost (USD)**
Gown and Gloves	19.79
Blade	0.94
Gauze	24.39
Normal Saline	3.53
Sucker	1.52
Diathermy	16.04
Sutures	21.99
Opsite Dressing	8.77
Hip Spica	128.34
Total Cost 1 (USD)	225.31
Femur Plate	93.58
Total Cost 2 (USD)	318.89

**Table III:** Surgeon’s and Anaesthetist Fees

**Operation (duration of surgery)**	**Surgeon’s Fee (USD)**	**Anaesthetist’s Fee (USD)**	**Operation Theatre Cost (USD)**	**Total Cost 3 (USD)**
Close Manipulative Reduction	207.67	69.22	53.48	330.37
(CMR) & Hip Spica (1 hour)				
Open Reduction only (2 hours)	454.81	151.60	106.95	713.37
Open Reduction & Femoral				
Shortening/Derotation (3 hours)	554.68	184.89	160.43	900
Open Reduction & Acetabuloplasty (3 hours)	554.68	184.89	160.43	900
Open Reduction, Femoral Shortening & Acetabuloplasty (4 hours)	650.94	216.98	213.90	1081.82

The admission costs included the cost of hospital stay and basic hematological and radiological investigations and medication (analgesic and antibiotic). The average hospital stay for the operation was three days (pre-operative, operative and post-operative). (Table IV)

**Table IV:** Admission cost [6-9]

**Admission**	**Cost (USD)**
Hospital Stay (3 days)	1.20
Basic Blood Investigation	13.37
(Full Blood Count, Group-cross Match)	
Pelvic Radiograph	16.04
Syrup Paracetamol	0.67
Parenteral Cefuroxime	6.70
Total Cost 4 (USD)	37.98

## Results

Out of the twenty-four DDH patients, only thirteen fulfilled the inclusion criteria. The mean age at time of presentation for treatment was 40.5 months: four children presented before six months, four presented between six to 24 months, and five at more than two years. Majority of them were unilateral DDH with left hip predominant. The principal presenting complaint was abnormal or limping gait.

All patients were female with three of them being first-born baby (23.07%), and three were breech intra-uterine presentation. None of them had a family history of DDH or antenatal oligohydramnios. Most of patients (61.53%) had one risk factor of DDH and the remaining had more than one risk factor (38.46%).

Four patients who presented early (less than six months old) were initially treated non-operatively with Pavlik harness or abduction splint; only one patient was successfully managed with Pavlik harness alone, the other three patients needed further intervention with closed manipulative reduction (CMR) and hip spica. Four patients who presented between 6 to 24 months of age, underwent open reduction; two of them were treated with CMR and hip spica earlier but failed. Majority of the patients who presented after two years of age underwent open reduction and femoral and acetabular procedures. The total procedures for the 3-year period of study were five CMR and hip spica, four open reduction alone, and four open reduction with femoral and acetabular osteotomy. Estimation of the surgical cost in this study are presented (Tables V and VI)

**Table V:** Estimation of the surgical cost in this study

**Operation**		**Total Cost (USD)**
Closed Manipulative Reduction (CMR) & Hip Spica	Hip Spica only + Total Cost 3 + Total Cost 4	496.69
Open Reduction only	Total Cost 1 + Total Cost 3 + Total Cost 4	976.66
Open Reduction & Femoral Shortening/Derotation	Total Cost 2 + Total Cost 3 + Total Cost 4	1,256.87
Open Reduction & Acetabuloplasty	Total Cost 1 + Total Cost 3 + Total Cost 4	1,163.29
Open Reduction, Femoral Shortening & Acetabuloplasty	Total Cost 2 + Total Cost 3 + Total Cost 4	1,438.69

**Table VI:** Cost estimation for early and late presentation of DDH

**Early (Presented at less than 6 month old)**			**Late (Presented at more than 6 month old)**		
Four Ultrasound Screening, Two Pavlik Harness,			Two CMR & Hip Spica, Four Open Reduction (Alone),		
Two Abduction Splint and Three CMR & Hip Spica			and Four Open Reduction with Bony Procedures		
	USD 1,730.73		USD 10,654.78
	(13.97% from Grand Total)		(86.03% from Grand Total)
	Grand Total: USD 12,385.51			

## Discussion

Developmental Dysplasia of Hip (DDH) is a spectrum of hip pathology that can present as hip subluxation or complete hip dislocation with dysplastic acetabulum. It can be detected early soon after delivery by clinical examination and ultrasound screening. Early screening proved to reduce the incident of delayed presentation of DDH^10^.

The definition of late presentation varies. Clarke *et al* defined the late presentation as any child presented with DDH more than 3 months of age^11^, whereas R Gul *et al* choose presentation more than of 6 months old are considered late presentation^10^. In this study we defined late presentation as the presentation for the first consultation at beyond 6 months of age.

We found the majority of patients in our series presented at age more than six months (69.23%), similar to the finding in the local study conducted in University of Malaya, in which, however, the age of 4-month was the index age for classification of early or late presentation of DDH^4^. Delayed presentation of DDH may cause higher morbidity and the outcomes of surgical treatment are unpredictable^12^.

From 2012 till 2014, the overall cost of management of DDH was about USD 12,385.51 [official Bank Negara (National Bank) exchange rate in June 2015 of USD 1= RM3.74] 86% of the amount was used to treat late presentation of DDH and most of this was the cost of surgery. The early presenting DDH was treated non-operatively with Pavlik harness or abduction splint before deciding on surgery, and the surgery (if necessary) usually was less complex that required less cost, as there was no bony procedure and/or implant usage. Furthermore, the operation time was shorter than the more complex surgery, thus the operation theatre cost was less. The later the DDH patient came for the first treatment, the more complex surgery was needed. Bony procedures with or without implant were more costly when compared to the open reduction alone. These also increased the patient’s morbidity with more pain and scars, and longer operation time for the child with greater risk of anaesthesia.

Neonatal screening programs may help to detect this hip problem as early as the birth of the baby. The programs do not only focus on examination of the baby but also help to increase awareness among parents regarding DDH with targeted ultrasound for those at risk. R Gul *et al* reported significant reduction of late presenting DDH after implementation of the screening programs. The surgical management was also changed from open to close techniques^10^. This will save fund that can be deviated to the other health and education sectors.

Apart from small number of cases, other limitation of this analysis is the estimated cost that only focused on the health care management of DDH. The true impact should include the socio-economic effect on working-parents who might need to take unpaid leave especially when their child is on 3-month hip spica follow-up.

In conclusion, the management of late presentation of DDH gives strong financial burden on the country. Substantial amount of money is spent to manage such delayed cases; thus early detection of DDH with systematic neonatal screening should be well planned and implemented in order to minimize the incidence of the late presenting DDH and subsequently reduce the economic burden to our government.
